# Hints and Improvements in the Practice of Medicine, Surgery, and Pharmacy

**Published:** 1800-03

**Authors:** 


### HINTS AND IMPROVEMENTS

### IN THE PRACTICE OF

### MEDICINE, SURGERY, AND PHARMACY.

---

*To the Editors of the Medical and Physical Journal.*

GENTLEMEN,

SHOULD the following notice to the Faculty appear worthy a place in your useful Journal, I should esteem myself obliged by its insertion.

I have the honour to remain,

GENTLEMEN,

Your obedient servant,

E. KENTISH, Surgeon.

*Newcastle upon Tyne, January,* 1800.

---

*To all Medical Practitioners who have had an Opportunity of observing the diseased Actions, arising from the Application of heated Substances to the living Body, commonly termed Burns, or Scalds.*

AS I have had several communications from my professional brethren since the publication of my Essay on the above subject, I wish to offer to the whole, an opportunity of recording their observations. To those who have aided the investigation, I thus publicly return my sincere thanks, and particularly to the philosophic author of Medicina Nautica. It gives me pleasure to report, that the faculty of this neighbourhood (for the most part) have adopted the practice recommended; and their opinion of its efficacy is not less flattering to me than the avowal of it is creditable to themselves. Mr. S. Hammmick, jun. of the Royal Naval Hospital at Plymouth, has favoured me with his opinion of its superior merit to every other means used in that extensive institution, where he has an ample field for experiment, from the frequent explosions of gunpowder on board his Majesty's ships. He says, "I am decidedly of opinion that the practice of applying immediately to burns the spirit

ſpirit of turpentine, is the beſt I have ever yet ſeen adopted, as the proceſs to ſuppuration is, in general, more rapid, and thoſe irregular marks, or ſeams, found after other applications, are not to be met with after the turpentine; neither is the ſkin ſo diſpoſed to crack, or break open again, as was formerly too often the caſe, producing the moſt troubleſome and irritable ſores." The leading features I would wiſh to point out, as forming the baſis of compariſon are, 1ſt. The length of period from the accident to an unequivocal ſuppuration, (*i. e.* in burns of ſimilar parts and extent); this will form a pretty exact criterion of the propriety of the treatment during the firſt ſtage. 2dly. From the point of the ſuppuration to the cure, which will enable us to judge of the treatment of the ſecond ſtage. The above are the two leading points; and, if the ſymptoms in the two ſtages are accurately marked, may include the whole treatment. But whatever obſervations I may be favoured with, I promiſe to record them, whether for or againſt the practice, as I diſclaim all ſelfiſh views, in a matter of ſuch importance. Thoſe who favour me with their communications, will addreſs them to Mr. Kentish, Surgeon, *Newcaſtle upon Tyne.*

